# Association of multidisciplinary perioperative enhanced recovery pathways with perioperative outcomes in pediatric scoliosis correction surgery: a systematic review and meta-analysis

**DOI:** 10.3389/fped.2026.1968212

**Published:** 2026-10-29

**Authors:** Ping Ma, Jianjun Lai

**Affiliations:** Surgical Clinic, Affiliated Provincial Hospital of Shandong First Medical University, Jinan City, China

**Keywords:** enhanced recovery, meta-analysis, pediatric spine, perioperative care, scoliosis

## Abstract

**Background:**

Multidisciplinary perioperative enhanced recovery pathways are increasingly utilized in pediatric scoliosis correction surgery, yet the robustness of their pooled outcome estimates compared to usual care remains inadequately characterized. This systematic review and meta-analysis evaluates the pathway-level associations of these protocols with both clinical and operational outcomes.

**Methods:**

We systematically searched PubMed, EMBASE, CINAHL, Scopus, Web of Science, and the Cochrane Library from inception through March 31, 2026. Eligible studies compared structured multidisciplinary pathways with usual care in pediatric scoliosis populations. Outcomes included postoperative complications, 30-day unplanned readmission, operative duration, and length of hospital stay. Outcome definitions and ascertainment were extracted as reported by the original studies, including the reported follow-up period and measurement reference point where available. Because postoperative complications could encompass clinically heterogeneous events and length of hospital stay could be influenced by institution-specific discharge criteria and measurement conventions, no *post hoc* uniform definition was imposed across studies; relevant differences in outcome ascertainment were considered when interpreting the pooled evidence. Pooled odds ratios (ORs) and weighted mean differences (WMDs) with 95% confidence intervals (CIs) were derived using random-effects models. Given the inherent methodological heterogeneity of real-world observational data, restricted maximum likelihood (REML) estimation with Knapp-Hartung adjustment was applied as a prespecified sensitivity analysis for outcomes demonstrating extreme statistical heterogeneity (I^2^ > 90%). DerSimonian-Laird was the primary analysis for all outcomes.

**Results:**

Twenty studies were included in the review, with different subsets contributing to each outcome-specific meta-analysis. Twelve studies (*n* = 1,874) revealed no statistically significant association with postoperative complications (OR: 0.75; 95% CI: 0.43 to 1.31; I^2^ = 34.5%). Seven studies (*n* = 1,386) demonstrated no statistically significant association with 30-day readmissions (OR: 1.21; 95% CI: 0.73 to 2.02; I^2^ = 0.0%). While the primary DerSimonian–Laird analysis showed a significant reduction in operative duration (WMD: −26.05 min; 95% CI: −48.54 to −3.55), this finding became statistically non-significant under the more conservative REML with Knapp–Hartung adjustment (WMD: −22.46 min; 95% CI: −59.23 to 14.32; *p* = 0.197). In contrast, fifteen studies demonstrated a statistically significant but uncertain association with length of hospital stay (WMD: −1.75 days; 95% CI: −2.18 to −1.32; *p* < 0.001), which remained robust after conservative adjustment (*p* < 0.001). Extreme heterogeneity (I^2^ > 95%) was observed for all continuous outcomes, and significant publication bias was detected for length of hospital stay (Egger's test, *p* = 0.003).

**Conclusion:**

This meta-analysis provides a quantitative signal that multidisciplinary enhanced recovery pathways are associated with a reduced length of hospital stay, suggesting an improvement in perioperative discharge efficiency. However, the evidence for reduced operative duration is statistically fragile and inconclusive, and no statistically significant associations were found for complications or readmissions. The overall certainty of the evidence is very low due to the predominance of retrospective designs, extreme heterogeneity, and publication bias.

## Introduction

Scoliosis correction surgery represents one of the most technically demanding procedures in pediatric spinal surgery, necessitated by the progressive nature of the deformity which, if left untreated, may significantly compromise cardiopulmonary function and impair quality of life (1). The optimization of perioperative care is contingent not merely upon the technical execution of the surgical procedure, but upon a coordinated multidisciplinary strategy addressing preoperative preparation, intraoperative homeostasis, and postoperative rehabilitation (2).

Multidisciplinary perioperative enhanced recovery pathways are composite bundles of care that synergistically integrate preoperative patient education, standardized intraoperative anesthesia and surgical workflows, multimodal analgesia protocols, early mobilization, and expedited discharge planning (3). While nursing staff frequently deliver and coordinate these activities, they are inherently embedded within broader pathway packages involving surgeons, anesthesiologists, physiotherapists, and institutional workflow changes. Therefore, the present review synthesizes pathway-level associations rather than the independent causal contribution of nursing care or any other individual component. Because the interventions were implemented as multidisciplinary bundles, the available comparative studies do not permit attribution of observed associations to a single discipline or pathway element, as this cannot be methodologically isolated (4).

Previous systematic reviews have broadly examined enhanced recovery after surgery (ERAS) protocols in spinal deformity populations, generally reporting shorter lengths of stay without increases in complication rates (5–8). However, several of these prior syntheses were constrained by a failure to robustly address extreme statistical heterogeneity using conservative estimators, nor did they explicitly account for potential publication bias or systematically assess the certainty of the cumulative evidence. Consequently, a rigorous quantitative synthesis that applies conservative statistical adjustments and evaluates evidence quality is urgently needed to guide clinical decision-making.

Therefore, the primary objective of this systematic review and meta-analysis is to quantitatively synthesize the pathway-level associations of structured multidisciplinary perioperative enhanced recovery protocols with postoperative complications, 30-day readmission rates, duration of surgery, and length of hospital stay in the pediatric scoliosis population, while employing rigorous methodological standards to ensure the robustness of the inferences.

## Methods

This systematic review and meta-analysis were conducted in strict accordance with the Preferred Reporting Items for Systematic Reviews and Meta-Analyses (PRISMA) 2020 guidelines (9). The study was not prospectively registered in PROSPERO and no publicly accessible protocol or registry record was available. The methodological framework, including the PICOS eligibility criteria, outcomes, search strategy, and planned evidence-synthesis approach, was established *a priori* before study selection. Any methodological decisions made during the review were documented and are described transparently in the present report.

The eligibility criteria were defined using a PICOS framework. The population included pediatric patients undergoing scoliosis correction surgery, predominantly adolescents with idiopathic scoliosis, although one included study enrolled patients with early-onset scoliosis. Because early-onset scoliosis may differ from adolescent idiopathic scoliosis in age, disease characteristics, surgical complexity, and perioperative requirements, this population was retained only when the intervention and surgical context met the predefined eligibility criteria; its presence should nevertheless be considered when interpreting the generalizability of the pooled evidence. The intervention was defined as a prospectively defined and institutionally standardized multidisciplinary perioperative pathway comprising at least two coordinated care components implemented across at least two perioperative phases (preoperative, intraoperative, or postoperative). Eligible pathways could include combinations of patient education, standardized anesthesia or surgical workflows, multimodal analgesia, early mobilization, nutritional or supportive care, and structured discharge planning. Single-component interventions without a broader coordinated pathway were excluded. Because the included interventions were bundled, the present review evaluates pathway-level associations rather than the independent effect of any individual component. The comparison consisted of conventional or standard institutional care, including historical controls when these were explicitly used by the original study. Because historical controls may differ from contemporary pathway cohorts in surgical, anesthesia, nursing, mobilization, and discharge practices, comparator heterogeneity and secular trends were considered important limitations when interpreting the pooled associations. The primary outcomes included postoperative complications, 30-day unplanned readmission rates, duration of surgery, and length of hospital stay. Postoperative complications were defined as any adverse event reported by the original studies, including major and minor complications, without a uniform severity threshold, because the included studies used heterogeneous definitions. 30-day readmission was defined as unplanned readmission within 30 days of surgery, as reported by the original studies. Eligible study designs comprised randomized controlled trials, quasi-experimental studies, or comparative observational cohorts. Peer-reviewed journal articles and eligible preprints identified through the predefined database search were considered, with preprints explicitly identified as non-peer-reviewed evidence in the study characteristics table.

We performed comprehensive systematic searches of PubMed/MEDLINE, EMBASE, CINAHL, Scopus, Web of Science, and the Cochrane Library from inception up to March 31, 2026. No language restrictions were applied. The search strategies combined Medical Subject Headings and free-text terms for concepts including “Scoliosis”, “Spinal Fusion”, “Enhanced Recovery After Surgery”, “ERAS”, and “fast-track”. The complete database-specific search strategies, including controlled vocabulary, free-text terms, Boolean operators, applied filters, and the final search dates, are provided in the Supplementary Appendix to permit reproducibility. In addition, manual screening of the reference lists of all included studies and prior systematic reviews was conducted to identify additional potentially eligible records. The preprint was identified through hand-searching and reference lists. Conference abstracts and unpublished studies were not systematically searched. Grey literature was not systematically searched. Only peer-reviewed journal articles and one eligible preprint identified through the predefined search were included.

The study selection process was conducted in duplicate. Title and abstract screening, as well as the subsequent full-text eligibility assessment, were performed independently by two reviewers. Any disagreements were resolved through consensus, and no third reviewer was required. Data extraction was also performed independently by the same two reviewers using a standardized, piloted extraction form. Extracted variables included study characteristics, patient demographics, intervention specifications, comparator characteristics, outcome definitions and ascertainment, and outcome data. During data extraction, potential overlap between cohorts was specifically considered because several studies originated from the same institutions or research groups. Potential overlap was assessed by comparing institutions, author groups, study or recruitment periods, sample sizes, intervention implementation periods, and reported cohort characteristics. Publications from the same institutional program were not assumed to represent independent cohorts solely because they had different publication years; cohort independence was considered before inclusion in outcome-specific syntheses. Authors of primary studies were contacted via email to obtain missing data when required. When missing data could not be obtained from authors, available data were used, and no imputation was performed. Studies with insufficient data for a given outcome were excluded from that outcome-specific meta-analysis. Most included studies reported unadjusted effect estimates. Where adjusted estimates were available, we used the unadjusted estimates to maintain consistency across studies. Methodological quality was appraised independently and in duplicate. No randomized controlled trials were identified; therefore, the Cochrane Risk of Bias 2 (RoB 2) tool was planned but not applied. Observational studies were appraised using the Risk of Bias in Non-Randomized Studies of Interventions (ROBINS-I) tool. The ROBINS-I domains most frequently contributing to moderate or high risk-of-bias judgments were confounding, selection of participants, and measurement of outcomes. For summary purposes, studies rated as “moderate” under ROBINS-I were categorized as moderate risk, whereas studies rated as “serious” or “critical” were categorized as high risk for sensitivity analyses (10, 11). Overall judgments were summarized as low, moderate, or high risk of bias for descriptive presentation.

All meta-analytical procedures were conducted using Stata software. Random-effects models were used for all outcomes because of anticipated clinical and methodological heterogeneity across studies. For dichotomous outcomes, pooled odds ratios with 95% confidence intervals were calculated using a random-effects inverse-variance model based on the DerSimonian-Laird estimator, where an odds ratio greater than 1.0 indicates higher odds in the enhanced recovery group compared with usual care, and an odds ratio less than 1.0 indicates lower odds. For continuous outcomes, pooled weighted mean differences with 95% confidence intervals were utilized, provided all included studies employed the same measurement scale. A continuity correction of 0.5 was applied to studies with zero cells in dichotomous analyses. Statistical heterogeneity was quantified using the Cochrane Q statistic and the I^2^ statistic. Because of the inherent diversity of real-world observational data, the Knapp-Hartung adjustment was applied as a prespecified sensitivity analysis to all continuous outcomes demonstrating extreme heterogeneity (I^2^ > 90%), which included duration of surgery and length of hospital stay. When discrepancies between the primary analysis and the conservative adjustment arose, priority was given to the more robust inference provided by the Knapp-Hartung adjustment. Subgroup analyses by study design and intervention type were prespecified in the protocol. Leave-one-out sensitivity analyses were prespecified to assess the influence of individual studies on the pooled estimates. Sensitivity analyses excluding studies at high risk of bias were prespecified. Publication bias was assessed visually using funnel plots and statistically via Egger's regression test when at least ten studies were available for a given outcome (12). A two-sided *p*-value of less than 0.05 denoted statistical significance. Finally, the overall certainty of the body of evidence for each outcome was assessed using the Grading of Recommendations Assessment, Development and Evaluation (GRADE) framework, which considers five domains: risk of bias, inconsistency, indirectness, imprecision, and publication bias. A Summary of Findings table was constructed to present the pooled effect estimates, heterogeneity, certainty of evidence, and reasons for downgrading for each primary outcome. The table follows the GRADE framework and includes the number of studies, number of patients, effect estimate with 95% confidence interval, I^2^ statistic, certainty rating, and the domains contributing to downgrading.

## Results

The initial database search identified 1,543 records. After the removal of 321 duplicates, 1,222 records were screened based on titles and abstracts. Of the 113 full-text articles assessed for eligibility, 93 were excluded due to non-relevant interventions, non-relevant outcomes, or insufficient extractable data. Ultimately, 20 studies were incorporated into the systematic review and meta-analysis, as detailed in Figure 1 (13–32).

**Figure 1:**
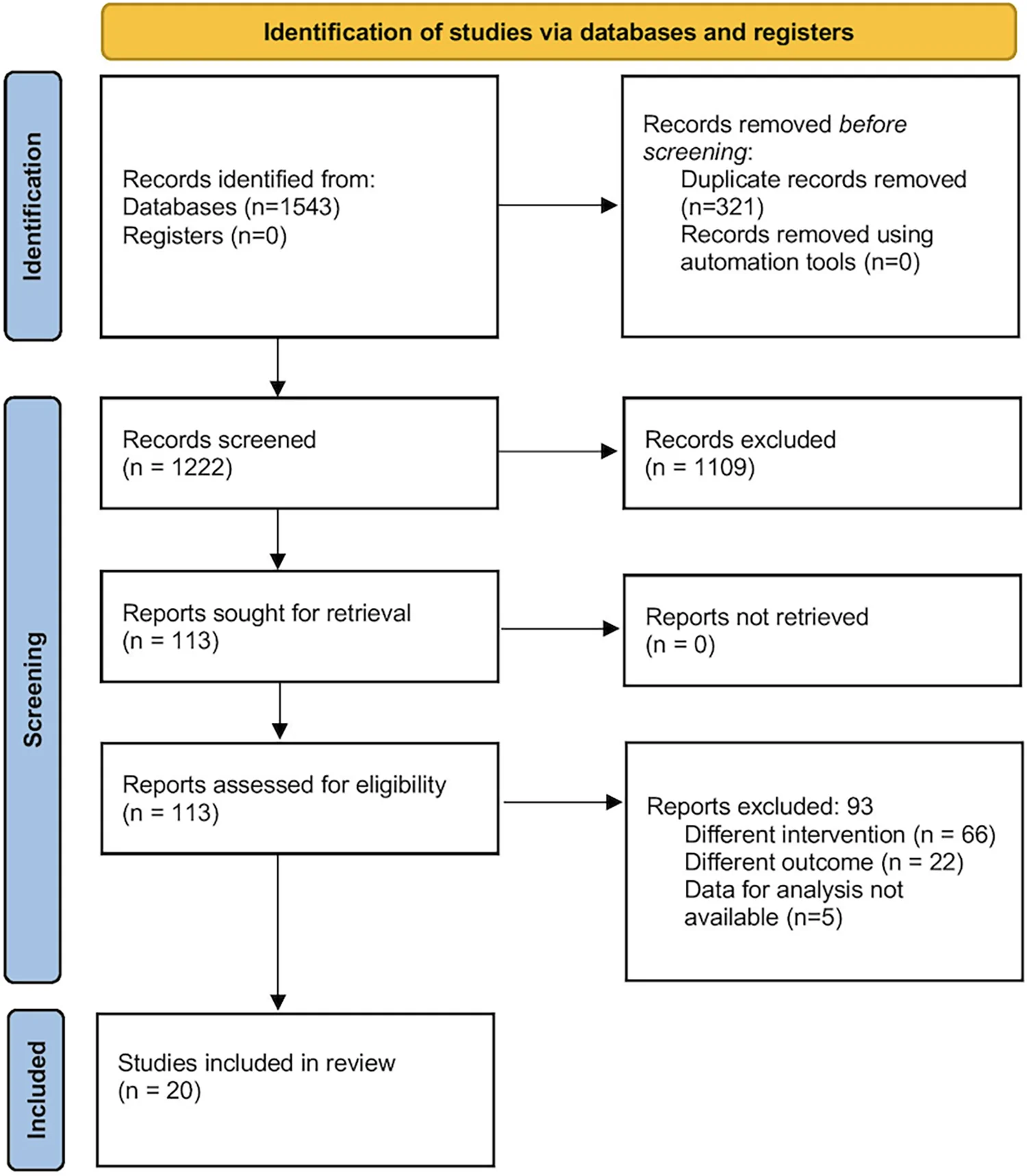
PRISMA 2020 flow diagram of study identification, screening, eligibility, and inclusion. A total of 1,543 records were identified from databases, 321 duplicates were removed, 1,222 records were screened, 113 full-text articles were assessed for eligibility, and 20 studies were included in the systematic review and meta-analysis.

The included populations were predominantly adolescents with idiopathic scoliosis undergoing posterior spinal fusion. The majority of the included studies were conducted in the United States, with additional contributions from China, Canada, France, Australia, and Denmark. Seventeen studies utilized retrospective cohort designs, while three were prospective. Based on the harmonized risk of bias assessment, six studies were classified as low risk, seven as moderate, and seven as high risk. The ROBINS-I domains most frequently contributing to moderate or high risk judgments were confounding, selection of participants, and measurement of outcomes. The comprehensive characteristics of the included cohorts, including study design, sample sizes, mean ages, and risk of bias judgments, are summarized in Table 1. A detailed mapping of each included study to intervention type and reported key components is provided in Supplementary Table S1.

**Table 1:** Characteristics of the included studies.

First Author & Year	Region	Study Design	Population	Intervention (n)	Control (n)	Age (Intervention, years)	Age (Control, years)	Risk of Bias	Intervention Type	Outcomes Contributed	Key Components
Barnett (13)	USA	Retrospective	AIS, PSF	48	44	14.4 ± 2.1	13.8 ± 1.7	High	ERAS	Complications; Readmissions	Preop education; multimodal analgesia; early mobilization; standardized discharge criteria
De Vries (14)	Canada	Retrospective	AIS, PSF	131	113	15.2 ± 2.0	15.3 ± 1.9	Moderate	Rapid Recovery	Complications; Readmissions; LOS	Preop education; opioid-sparing analgesia; early mobilization; early feeding; accelerated discharge planning
Ding (15)	China	Retrospective	AIS, PSF	45	45	15.36 ± 1.33	15.35 ± 1.53	Moderate	ERAS	Duration; LOS	Preop counseling; standardized anesthesia; multimodal analgesia; early mobilization; standardized discharge criteria
Fletcher (16)	USA	Retrospective	AIS, PSF	154	125	14.4 ± 1.9	14.7 ± 2.3	Low	Accelerated Discharge	Complications; Duration; LOS	Early discharge criteria; standardized postoperative pathway; caregiver education; outpatient follow-up
Fletcher (17)	USA	Retrospective	AIS, PSF	105	45	14.1 ± 1.6	14.3 ± 1.8	High	Accelerated Discharge	Complications; Duration	Early discharge criteria; standardized postoperative pathway; caregiver education; outpatient follow-up
Fletcher (18)	USA	Prospective	AIS, PSF	20	37	14.3 ± 2.1	16.09 ± 2.1	Low	ERAS	Complications; Duration; LOS	Preop counseling; standardized anesthesia; multimodal analgesia; early mobilization; standardized discharge criteria
Gornitzky (19)	USA	Retrospective	AIS, PSF	58	80	14.8 ± 2.3	15.0 ± 2.3	High	Rapid Recovery	Readmissions; LOS	Preop education; opioid-sparing analgesia; early mobilization; early feeding; accelerated discharge planning
Jeandel (20)	France	Retrospective	AIS, PSF	30	30	14.7 ± 1.25	14.5 ± 1.75	Moderate	ERAS	Complications; LOS	Preop counseling; standardized anesthesia; multimodal analgesia; early mobilization; standardized discharge criteria
Jian (21) [Preprint (not peer-reviewed)]	China	Retrospective	Early-Onset Scoliosis	35	35	13.17 ± 1.44	13.86 ± 1.50	Moderate	ERAS	LOS	Preop counseling; standardized anesthesia; multimodal analgesia; early mobilization; standardized discharge criteria
Kim (22)	USA	Prospective	AIS, PSF	36	36	14.4 ± 2.5	13.9 ± 2.0	Low	ERAS	Complications; Duration; LOS	Perioperative surgical home; multidisciplinary coordination; multimodal analgesia; early mobilization
Lebel (23)	Canada	Retrospective	AIS, PSF	59	81	14.1 ± 1.8	15.0 ± 1.8	High	ERAS	Complications; LOS	Preop counseling; standardized anesthesia; multimodal analgesia; early mobilization; standardized discharge criteria
Sanders (24)	USA	Retrospective	AIS, PSF	90	194	14.3	14.0	Low	Accelerated Discharge	Complications; Readmissions; LOS	Early discharge criteria; standardized postoperative pathway; caregiver education; outpatient follow-up
Spisak (25)	USA	Retrospective	AIS, PSF	20	22	14.0 ± 1.8	14.6 ± 1.5	High	ERAS	Duration; LOS	Preop counseling; standardized anesthesia; multimodal analgesia; early mobilization; standardized discharge criteria
Temby (26)	Australia	Retrospective	AIS, PSF	61	32	14.0 ± 1.6	15.4 ± 1.5	Moderate	ERAS	Complications; Readmissions; LOS	Preop counseling; standardized anesthesia; multimodal analgesia; early mobilization; standardized discharge criteria
Tøndevold (27)	Denmark	Retrospective	AIS, PSF	88	66	14.0 ± 1.25	14.0 ± 0.8	Moderate	Accelerated Discharge	Duration; LOS	Early discharge criteria; standardized postoperative pathway; caregiver education; outpatient follow-up
Yang (28)	China	Retrospective	AIS, PSF	35	44	14.6 ± 2.0	14.5 ± 2.1	Moderate	ERAS	Duration; LOS	Preop counseling; standardized anesthesia; multimodal analgesia; early mobilization; standardized discharge criteria
Rao (29)	USA	Retrospective	AIS, PSF	139	51	14.9 ± 1.5	13.5 ± 2.25	High	Rapid Recovery	Complications; Duration	Preop education; opioid-sparing analgesia; early mobilization; early feeding; accelerated discharge planning
Oetgen (30)	USA	Retrospective	AIS, PSF	169	44	N/R	N/R	Low	ERAS	Complications; Readmissions	Lean process mapping; standardized orders; multidisciplinary coordination; early mobilization
Muhly (31)	USA	Retrospective	AIS, PSF	84	238	N/R	N/R	High	Rapid Recovery	Readmissions	Preop education; opioid-sparing analgesia; early mobilization; early feeding; accelerated discharge planning
Chan (32)	China	Prospective	AIS, PSF	74	33	N/R	N/R	Low	Rapid Recovery	LOS	Preop education; multimodal analgesia; early mobilization; early feeding; accelerated discharge planning

AIS, adolescent idiopathic scoliosis; PSF, posterior spinal fusion; ERAS, enhanced recovery after surgery; LOS, length of hospital stay; N/R, not reported.

For the outcome of postoperative complications, a total of 12 studies encompassing 1,874 patients contributed to the pooled analysis. The random-effects meta-analysis revealed no statistically significant difference in complication rates between the multidisciplinary enhanced recovery pathways and usual care (OR: 0.75; 95% CI: 0.43 to 1.31; *p* = 0.315), as demonstrated in Figure 2. The degree of heterogeneity was moderate (I^2^ = 34.5%). Visual inspection of the funnel plot revealed no distinct asymmetry, which was corroborated by a non-significant Egger's test (*p* = 0.91). Subgroup analyses based on study design and intervention type revealed no significant between-group differences, and a sensitivity analysis restricted to studies categorized as low or moderate risk of bias did not materially alter the effect estimate. The results of these additional analyses, including the leave-one-out sensitivity analysis, are presented in Supplementary Figures S1–S5.

**Figure 2:**
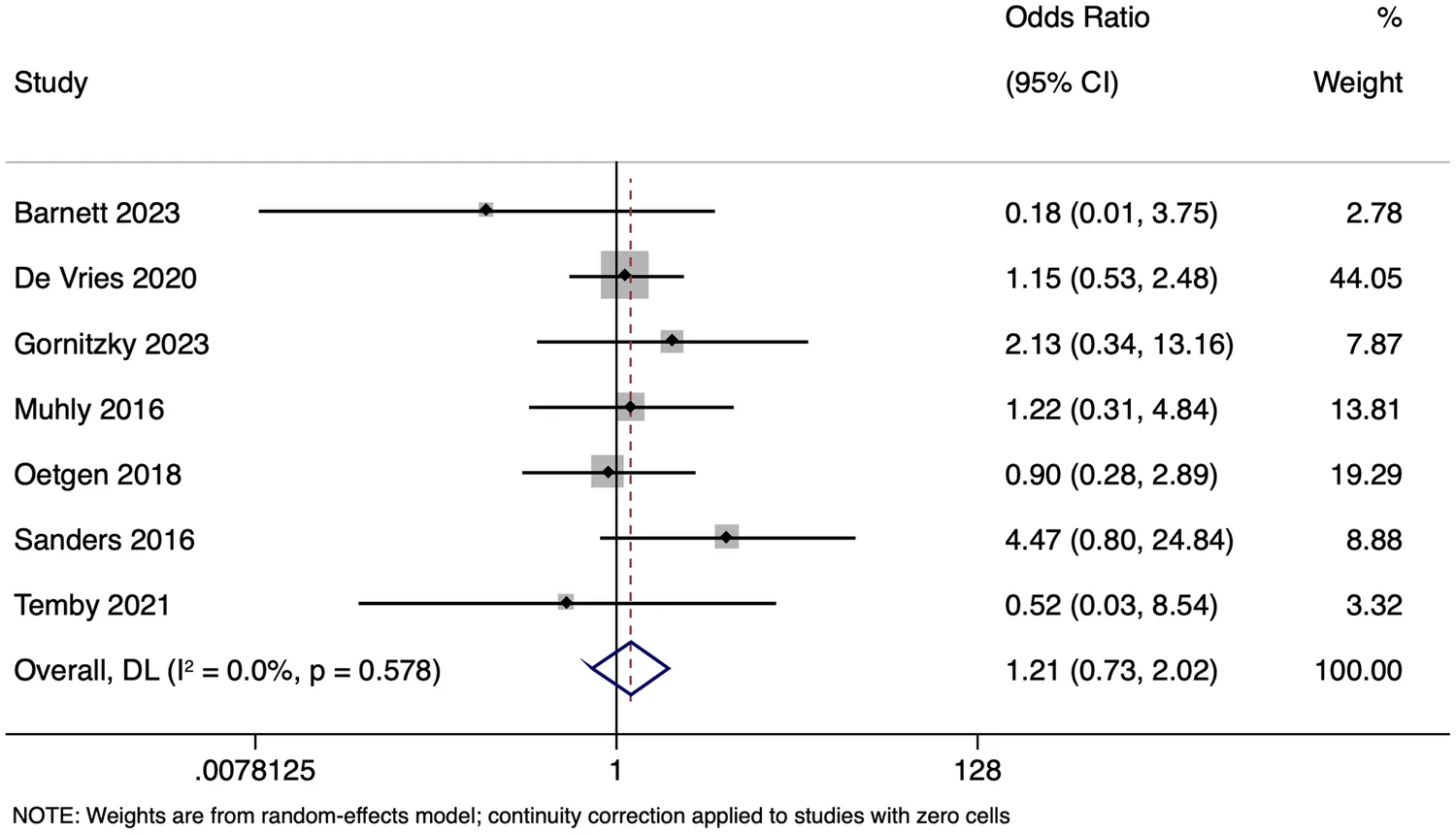
Forest plot of postoperative complications comparing multidisciplinary perioperative enhanced recovery pathways versus usual care. Pooled odds ratios (OR) with 95% confidence intervals (CIs) are shown. The overall pooled OR was 0.75 (95% CI: 0.43 to 1.31; I^2^ = 34.5%), indicating no statistically significant association.

Regarding 30-day readmission rates, the meta-analysis of seven studies comprising 1,386 patients demonstrated no statistically significant association between pathway implementation and readmission rates (OR: 1.21; 95% CI: 0.73 to 2.02; *p* = 0.47; OR > 1 numerically favors usual care, though the confidence interval includes 1.0), as shown in Figure 3. Heterogeneity was negligible across these studies (I^2^ = 0.0%*)* Subgroup analyses by intervention type and sensitivity analyses excluding high-risk studies confirmed the primary finding, with corresponding visuals provided in Supplementary Figures S6–S8.

**Figure 3:**
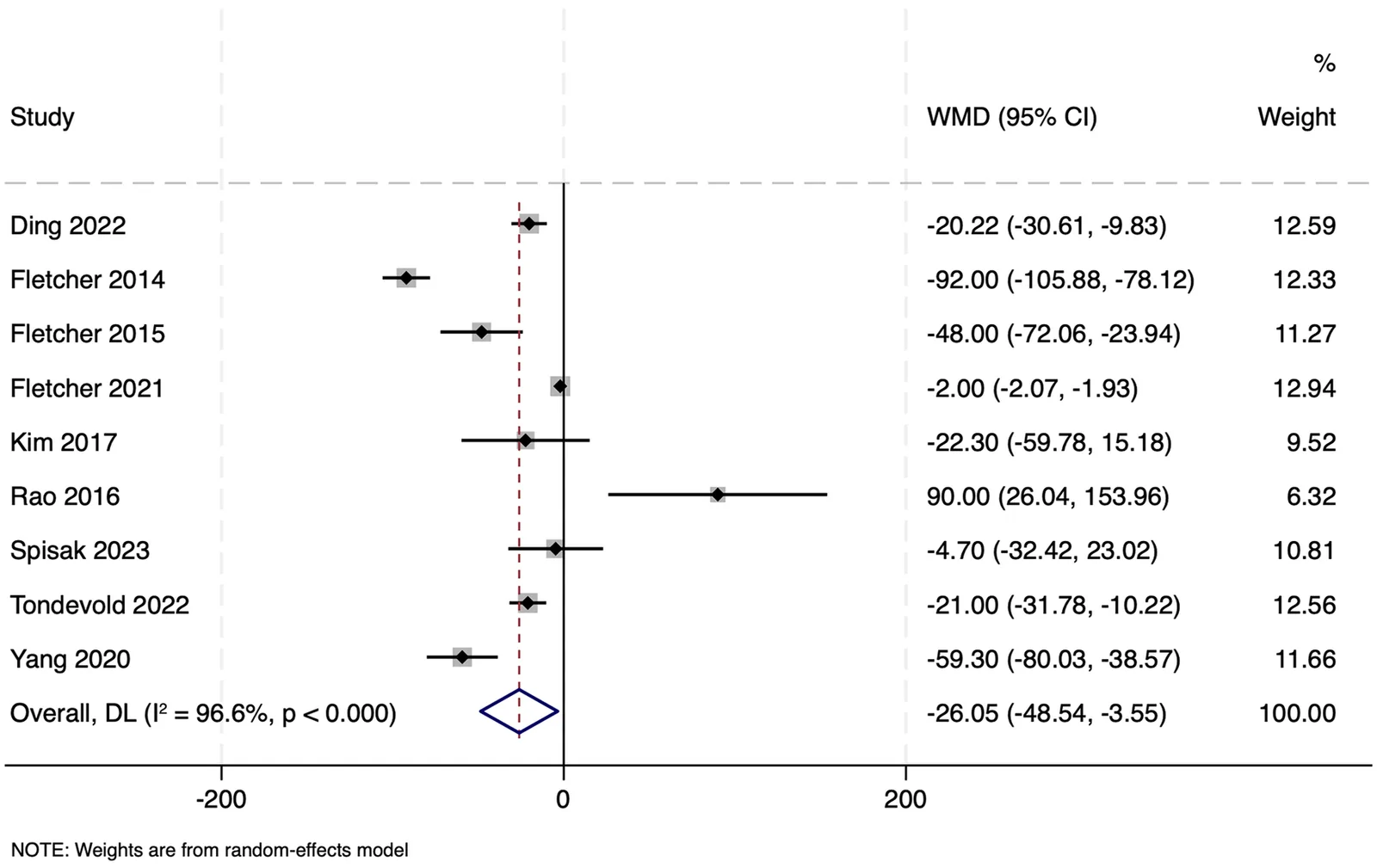
Forest plot of 30-day unplanned readmission comparing multidisciplinary perioperative enhanced recovery pathways versus usual care. Pooled odds ratios (OR) with 95% confidence intervals (CIs) are shown. The overall pooled OR was 1.21 (95% CI: 0.73 to 2.02; I^2^ = 0.0%), indicating no statistically significant association.

For the duration of surgery, the primary DerSimonian-Laird random-effects meta-analysis of nine studies with 1,113 patients indicated a statistically significant reduction in operative time among patients treated within enhanced recovery pathways (WMD: −26.05 min; 95% CI: −48.54 to −3.55; *p* = 0.023), as illustrated in Figure 4. However, this was accompanied by extreme statistical heterogeneity (I^2^ = 96.6%). Stratified analysis showed a significant association only in retrospective cohorts, while prospective studies yielded a non-significant estimate. Because of the extreme variance, we applied the pre-specified conservative REML with Knapp–Hartung sensitivity analysis. Under this robust statistical framework, the association between enhanced pathways and shorter operative duration became statistically non-significant (WMD: −22.46 min; 95% CI: −59.23 to 14.32; *p* = 0.197). Thus, while the direction of the effect remained favorable, the statistical uncertainty introduced by the profound heterogeneity renders the finding statistically inconclusive. Detailed subgroup analyses, leave-one-out analyses, and sensitivity analyses for this outcome are provided in Supplementary Figures S9–S12.

**Figure 4:**
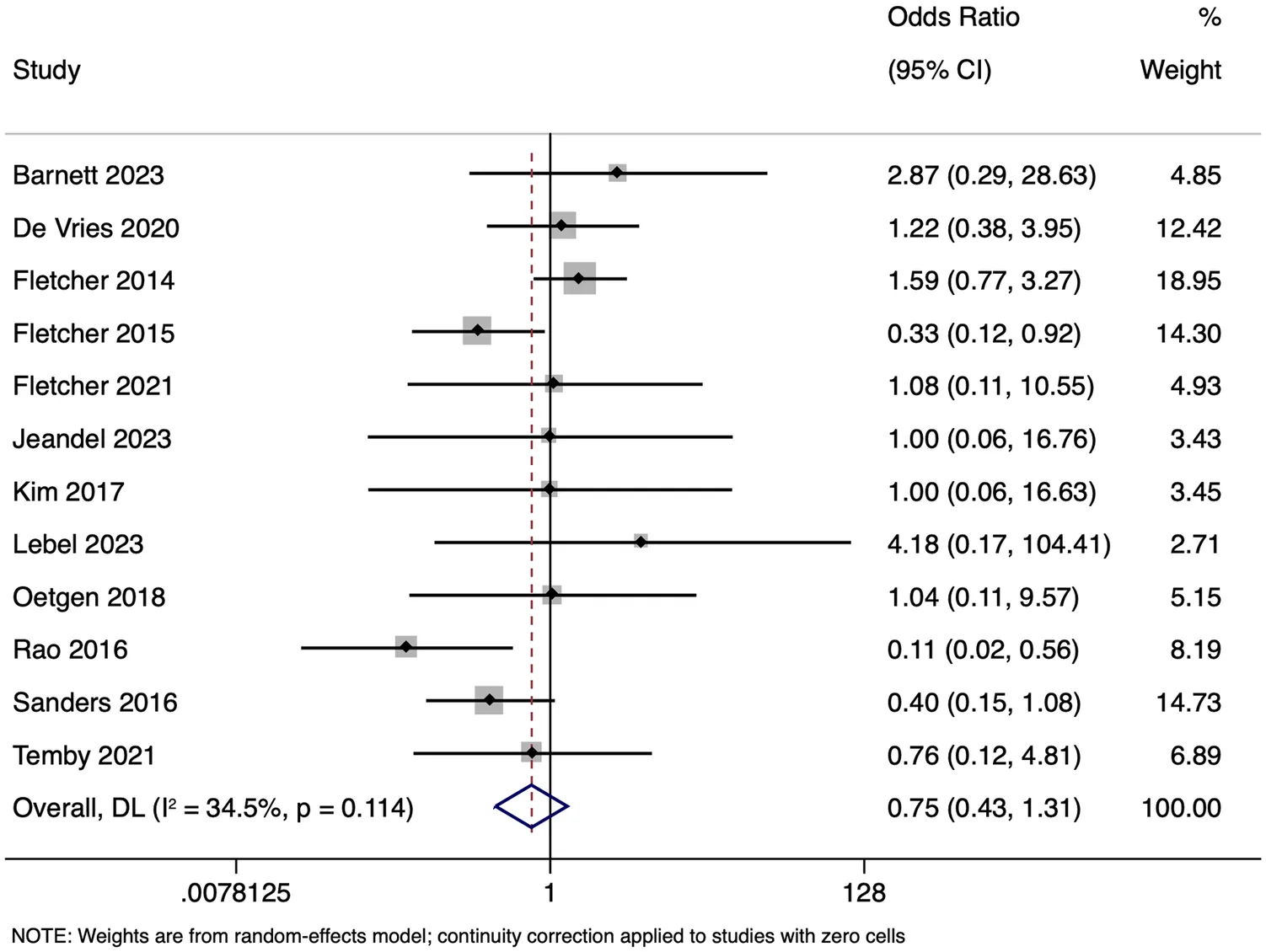
Forest plot of duration of surgery in minutes comparing multidisciplinary perioperative enhanced recovery pathways versus usual care. Pooled weighted mean differences (WMD) with 95% confidence intervals (CIs) are shown. Negative values favor enhanced recovery pathways. The overall pooled WMD was −26.05 min (95% CI: −48.54 to −3.55; I^2^ = 96.6%). This finding became statistically non-significant after the conservative REML with Knapp-Hartung adjustment.

For length of hospital stay, fifteen studies comprising 1,909 patients contributed to the pooled estimate. The enhanced recovery pathways were associated with a significant reduction in hospitalization duration (WMD: −1.75 days; 95% CI: −2.18 to −1.32; *p* < 0.001), as presented in Figure 5. Heterogeneity was exceptionally high (I^2^ = 97.3%). Subgroup analyses demonstrated consistent reductions across all study designs and intervention subtypes. Notably, visual inspection of the funnel plot revealed significant asymmetry, and Egger's regression test was statistically significant (*p* = 0.003), indicating funnel-plot asymmetry consistent with possible small-study effects. Because funnel-plot asymmetry may arise from publication bias, small-study effects, heterogeneity, or other methodological differences, this finding should not be interpreted as definitive evidence of publication bias. Nevertheless, the association remained statistically robust under the conservative REML with Knapp–Hartung adjustment (WMD: −1.80 days; 95% CI: −2.46 to −1.15; *p* < 0.001). While the length of hospital stay reduction appears more robust than the operative duration finding, the precise magnitude should be interpreted cautiously due to the observed publication bias and residual heterogeneity. The comprehensive subgroup and sensitivity analyses, as well as the publication bias assessment for this outcome, are detailed in Supplementary Figures S13–S17.

**Figure 5:**
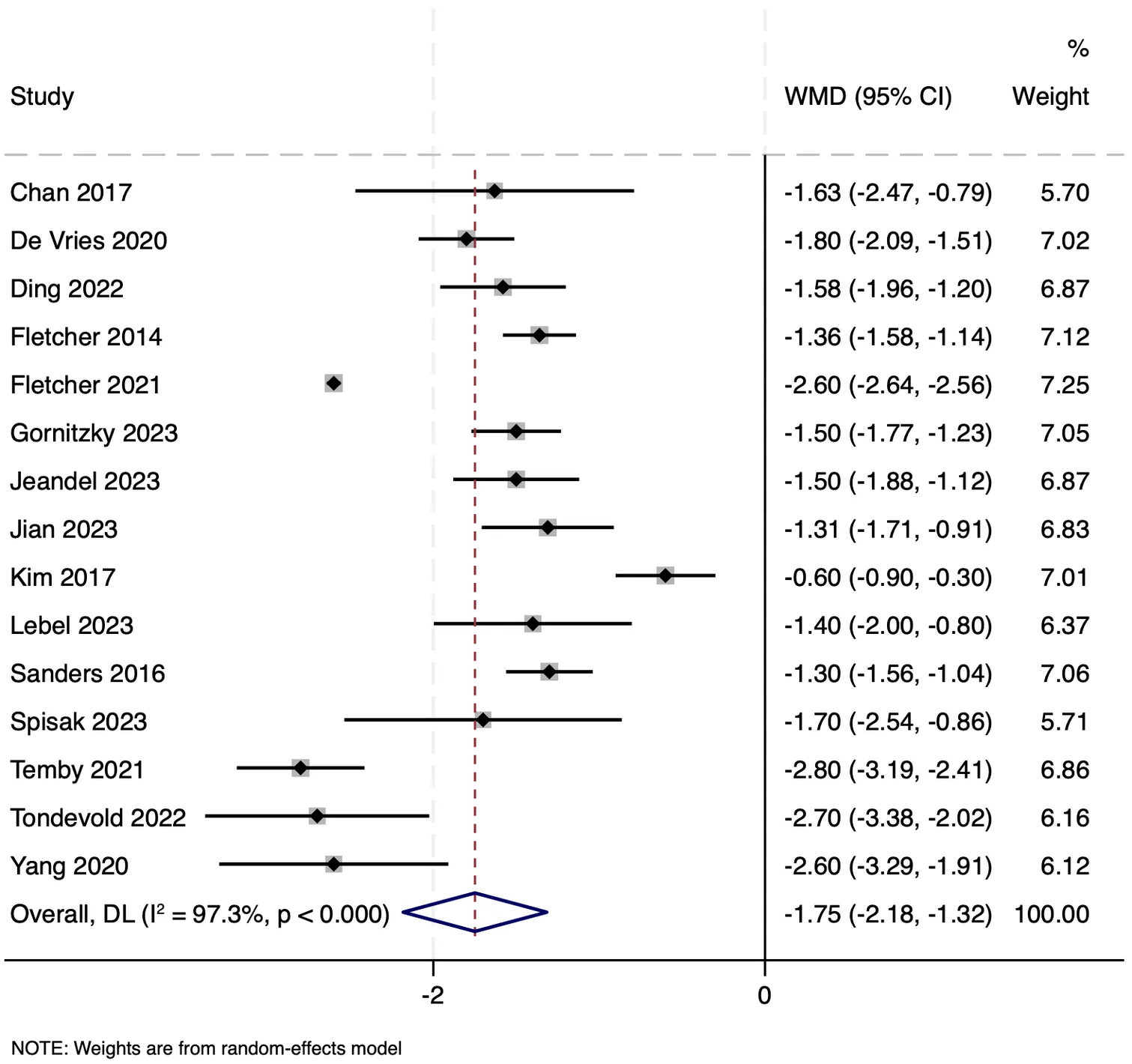
Forest plot of length of hospital stay in days comparing multidisciplinary perioperative enhanced recovery pathways versus usual care. Pooled weighted mean differences (WMD) with 95% confidence intervals (CIs) are shown. Negative values favor enhanced recovery pathways.

Using the GRADE framework, the certainty of evidence was rated as very low for all four outcomes. Downgrading was principally related to the predominance of retrospective observational studies and the resulting risk of bias and residual confounding; inconsistency was also an important concern for the continuous outcomes because of substantial between-study heterogeneity. For length of hospital stay, funnel-plot asymmetry and the possibility of small-study effects provided an additional concern. The individual reasons for downgrading are summarized in Table 2 (Summary of Findings). This rating was primarily driven by the overwhelming reliance on retrospective observational designs, which introduces significant risk of bias and residual confounding. In addition, the extreme unexplained inconsistency for continuous outcomes and the documented publication bias for length of hospital stay contributed to the downgrading of the evidence certainty.

**Table 2:** Summary of findings (GRADE) for multidisciplinary enhanced recovery pathways vs. Usual Care in Pediatric Scoliosis Correction Surgery.

Outcome	Studies (n)	Patients (n)	Effect Estimate (95% CI)	I^2^	Certainty (GRADE)	Reasons for Downgrading
Postoperative complications	12	1,874	OR 0.75 (0.43 to 1.31)	34.5%	Very low	Risk of bias; indirectness; imprecision
30-day readmission	7	1,386	OR 1.21 (0.73 to 2.02)	0.0%	Very low	Risk of bias; imprecision
Duration of surgery	9	1,113	WMD −26.05 min (−48.54 to −3.55); non-significant after Knapp–Hartung adjustment (WMD −22.46 min; −59.23 to 14.32)	96.6%	Very low	Risk of bias; inconsistency; imprecision
Length of hospital stay	15	1,909	WMD −1.75 days (−2.18 to −1.32); robust after Knapp–Hartung adjustment (WMD −1.80 days; −2.46 to −1.15)	97.3%	Very low	Risk of bias; inconsistency; publication bias/small-study effects

OR, odds ratio; WMD, weighted mean difference; CI, confidence interval; GRADE, Grading of Recommendations Assessment, Development and Evaluation.

## Discussion

This meta-analysis synthesized the available comparative evidence regarding multidisciplinary perioperative enhanced recovery pathways in pediatric scoliosis correction surgery. Our findings provide a quantitative signal suggesting that such pathways are associated with a statistically significant but uncertain reduction in length of hospital stay, while the evidence supporting a reduction in operative duration is rendered statistically fragile after correction for extreme heterogeneity. Furthermore, no statistical associations were observed for postoperative complications or 30-day readmissions.

The pooled analysis was associated with a shorter length of hospital stay; however, interpretation of the magnitude requires caution because statistical heterogeneity was very high and the included pathways differed in their discharge criteria and institutional practices. The estimate should therefore be viewed as an average association across heterogeneous settings rather than a uniform reduction expected in all program (5–7). However, our findings diverge from the ERAS literature in adult general surgery, where reduced complication rates are commonly reported (33). This discrepancy may be attributable to the unique complexity of spinal deformity correction; major perioperative complications are heavily contingent upon the extent of osteotomies, the number of levels fused, and neuromonitoring challenges, which are less modifiable by perioperative protocols alone (34). Similarly, the absence of a significant effect on readmissions contrasts with total joint arthroplasty literature. In the context of pediatric scoliosis, 30-day readmissions are often driven by delayed surgical site infections or hardware failure, complications that are inherently surgical and less susceptible to discharge planning interventions (35, 36).

Regarding perioperative efficiency, our interpretation warrants significant caution. The extreme heterogeneity (I^2^ > 96%*)* and the loss of statistical significance for operative duration following the Knapp–Hartung adjustment highlight the fragility of this finding, as operative time is profoundly influenced by institutional volume, surgeon experience, and the complexity of the curve. In contrast, while the reduction in length of hospital stay remained statistically robust under the conservative adjustment, this endpoint frequently serves as a surrogate marker of discharge efficiency rather than a pure metric of biological recovery. Many of the included pathways deliberately accelerated discharge criteria and incorporated structured discharge planning as active components of the intervention. Therefore, the observed reduction reflects a synergistic effect of clinical recovery and administrative discharge protocols, rather than necessarily indicating a substantially accelerated physiological healing process (37, 38). This distinction is critical for clinicians interpreting the results.

The principal limitations of this study are intrinsic to the primary literature. Substantial clinical and methodological heterogeneity was also present across the included studies, including differences in study design, institutional setting, scoliosis population, pathway composition, comparator practice, and outcome definitions. Therefore, the pooled estimates should be interpreted as average pathway-level associations across heterogeneous settings rather than as uniform effects applicable to every pediatric scoliosis program. Furthermore, the dominance of retrospective designs in the evidence base inevitably introduces selection bias and confounding by indication, and secular trends in surgical or anesthesia practices may have influenced outcomes. The observed funnel-plot asymmetry for length of hospital stay raises the possibility of small-study effects or publication-related mechanisms and may affect the precision and magnitude of the pooled estimate. Importantly, the independent causal contribution of individual disciplines, particularly nursing, could not be isolated because the interventions were inherently bundled. The review was not prospectively registered in PROSPERO, which may introduce reporting bias; however, the protocol was established *a priori*. Consequently, these findings must be interpreted as pathway-level associations rather than causal evidence for any specific component. Finally, the “very low” certainty of evidence according to the GRADE framework indicates that future high-quality research is highly likely to change these estimates.

The primary strengths of this review include a rigorous, systematic methodology adhering to strict reporting guidelines, comprehensive multi-database searching, dual independent review processes, and the implementation of conservative statistical adjustments to mitigate the influence of extreme variance. Future research should prioritize prospective, multicenter designs with factorial or stepped-wedge frameworks that allow for the disambiguation of specific intervention components. Additionally, consistent reporting of standard ERAS elements, long-term functional outcomes, and health-economic evaluations is urgently needed to define the true clinical utility of these protocols.

## Conclusion

In conclusion, this meta-analysis provides a quantitative signal that enhanced recovery pathways are associated with a statistically significant but uncertain reduction in length of hospital stay, likely overestimated due to publication bias and extreme heterogeneity. Evidence for reduced operative duration is inconclusive, and no statistically significant associations were found for complications or 30-day readmissions. Given the very low certainty of evidence, pooled estimates should be viewed as indicators of perioperative efficiency rather than precise benchmarks, with implementation tailored to local resources and monitored for unforeseen consequences.

## Data Availability

The original contributions presented in the study are included in the article/Supplementary Material, further inquiries can be directed to the corresponding author/s.

## Supplementary material

Supplementary Figure S1 Funnel plot assessing publication bias for postoperative complications. The solid vertical line represents the pooled effect estimate, and the dashed lines represent pseudo 95% confidence intervals. Visual inspection revealed no distinct asymmetry, consistent with the non-significant Egger's test. Note: if the provided funnel plot has an x-axis from 0 to 20, please replace it with the correct funnel plot for odds ratios, as the current axis is not compatible with an OR of 0.75.

Supplementary Figure S2 Subgroup forest plot of postoperative complications stratified by study design. Pooled odds ratios (OR) with 95% confidence intervals (CIs) are presented separately for retrospective and prospective studies.

Supplementary Figure S3 Subgroup forest plot of postoperative complications stratified by intervention type. Pooled odds ratios (OR) with 95% confidence intervals (CIs) are presented separately for ERAS protocols, rapid recovery pathways, and accelerated discharge pathways.

Supplementary Figure S4 Leave-one-out sensitivity analysis for postoperative complications. The figure shows the pooled odds ratio (OR) after sequential omission of each included study to assess the influence of individual studies on the overall estimate.

Supplementary Figure S5 Sensitivity analysis for postoperative complications after exclusion of studies at high risk of bias. Pooled odds ratios (OR) with 95% confidence intervals (CIs) are presented using a random-effects model.

Supplementary Figure S6 Subgroup forest plot of 30-day readmission rates stratified by intervention type. Pooled odds ratios (OR) with 95% confidence intervals (CIs) are presented separately for ERAS protocols and rapid recovery pathways.

Supplementary Figure S7 Leave-one-out sensitivity analysis for 30-day readmission rates. The figure shows the pooled odds ratio (OR) after sequential omission of each included study.

Supplementary Figure S8 Sensitivity analysis for 30-day readmission rates after exclusion of studies at high risk of bias. Pooled odds ratios (OR) with 95% confidence intervals (CIs) are presented using a random-effects model.

Supplementary Figure S9 Subgroup forest plot of duration of surgery stratified by study design. Pooled weighted mean differences (WMD) with 95% confidence intervals (CIs) are presented separately for retrospective and prospective studies. Negative values favor enhanced recovery pathways.

Supplementary Figure S10 Subgroup forest plot of duration of surgery stratified by intervention type. Pooled weighted mean differences (WMD) with 95% confidence intervals (CIs) are presented separately for ERAS protocols and accelerated discharge pathways. Negative values favor enhanced recovery pathways.

Supplementary Figure S11 Leave-one-out sensitivity analysis for duration of surgery. The figure shows the pooled weighted mean difference (WMD) after sequential omission of each included study. Negative values favor enhanced recovery pathways.

Supplementary Figure S12 Sensitivity analysis for duration of surgery after exclusion of studies at high risk of bias. Pooled weighted mean differences (WMD) with 95% confidence intervals (CIs) are presented using a random-effects model. Negative values favor enhanced recovery pathways.

Supplementary Figure S13 Funnel plot assessing publication bias for length of hospital stay. The solid vertical line represents the pooled effect estimate, and the dashed lines represent pseudo 95% confidence intervals. Visual asymmetry was observed, consistent with the significant Egger's test reported in the text.

Supplementary Figure S14 Leave-one-out sensitivity analysis for duration of surgery. The figure shows the pooled weighted mean difference (WMD) after sequential omission of each included study. Negative values favor enhanced recovery pathways.

Supplementary Figure S15 Subgroup forest plot of length of hospital stay stratified by intervention type. Pooled weighted mean differences (WMD) with 95% confidence intervals (CIs) are presented separately for ERAS protocols, rapid recovery pathways, and accelerated discharge pathways. Negative values favor enhanced recovery pathways.

Supplementary Figure S16 Leave-one-out sensitivity analysis for length of hospital stay. The figure shows the pooled weighted mean difference (WMD) after sequential omission of each included study. Negative values favor enhanced recovery pathways.

Supplementary Figure S17 Sensitivity analysis for length of hospital stay after exclusion of studies at high risk of bias. Pooled weighted mean differences (WMD) with 95% confidence intervals (CIs) are presented using a random-effects model. Negative values favor enhanced recovery pathways.
