# Supplementary material for: Association of multidisciplinary perioperative enhanced recovery pathways with perioperative outcomes in pediatric scoliosis correction surgery: a systematic review and meta-analysis
Source: Front Pediatr. 2026 Oct 29;14:1968212. doi: 10.3389/fped.2026.1968212 (PMC13755327; doi:10.3389/fped.2026.1968212)
Supplement: Supplementary file 1 [file Datasheet1.pdf]

Supplementary Appendix:

Search Strategy:

PubMed:

(

"Scoliosis"[Mesh]

OR scoliosis[tiab]

OR "idiopathic scoliosis"[tiab]

OR "adolescent idiopathic scoliosis"[tiab]

OR AIS[tiab]

OR "early onset scoliosis"[tiab]

OR "neuromuscular scoliosis"[tiab]

OR "congenital scoliosis"[tiab]

)

AND

(

"Spinal Fusion"[Mesh]

OR "Orthopedic Procedures"[Mesh]

OR "spinal fusion"[tiab]

OR "posterior spinal fusion"[tiab]

OR PSF[tiab]

OR "scoliosis correction"[tiab]

OR "corrective surgery"[tiab]

OR "scoliosis surgery"[tiab]

OR "deformity correction"[tiab]

OR "spine surgery"[tiab]

OR "posterior fusion"[tiab]

OR instrumentation[tiab]

)

AND

(

"Enhanced Recovery After Surgery"[tiab]

OR ERAS[tiab]

OR "enhanced recovery"[tiab]

OR "enhanced recovery pathway"[tiab]

OR "enhanced recovery protocol"[tiab]

OR "enhanced recovery program"[tiab]

OR "rapid recovery"[tiab]

OR "rapid recovery pathway"[tiab]

OR "rapid recovery protocol"[tiab]

OR "accelerated recovery"[tiab]

OR "accelerated recovery protocol"[tiab]

OR "accelerated discharge"[tiab]

OR "accelerated discharge pathway"[tiab]

OR "clinical pathway"[tiab]

OR "care pathway"[tiab]

OR "critical pathway"[tiab]

OR "perioperative pathway"[tiab]

OR "perioperative protocol"[tiab]

OR "perioperative surgical home"[tiab]

OR PSH[tiab]

OR "multimodal pathway"[tiab]

OR "multidisciplinary pathway"[tiab]

OR "standardized perioperative care"[tiab]

OR "standardized pathway"[tiab]

OR "fast track"[tiab]

OR "fast-track"[tiab]

OR "fast track surgery"[tiab]

)

AND

(

"Perioperative Care"[Mesh]

OR "Perioperative Nursing"[Mesh]

OR "Nursing Care"[Mesh]

OR "Patient Care Planning"[Mesh]

OR "Postoperative Care"[Mesh]

OR "Preoperative Care"[Mesh]

OR perioperative[tiab]

OR preoperative[tiab]

OR postoperative[tiab]

OR intraoperative[tiab]

OR nurse*[tiab]

OR nursing[tiab]

OR "nursing care"[tiab]

OR "perioperative nursing"[tiab]

OR "patient education"[tiab]

OR "pain management"[tiab]

OR "multimodal analgesia"[tiab]

OR mobilization[tiab]

OR "early mobilization"[tiab]

OR ambulation[tiab]

OR nutrition[tiab]

OR feeding[tiab]

OR discharge[tiab]

OR "discharge planning"[tiab]

OR "catheter removal"[tiab]

OR "drain removal"[tiab]

OR rehabilitation[tiab]

)

AND

(

"Postoperative Complications"[Mesh]

OR "Patient Readmission"[Mesh]

OR "Length of Stay"[Mesh]

OR "Surgical Procedures, Operative/time"[Mesh]

OR complication*[tiab]

OR readmission*[tiab]

OR "length of stay"[tiab]

OR LOS[tiab]

OR "hospital stay"[tiab]

OR "operative time"[tiab]

OR "operating time"[tiab]

OR "surgical time"[tiab]

OR "duration of surgery"[tiab]

OR fusion[tiab]

OR "fusion level"[tiab]

OR "levels fused"[tiab]

OR "spinal fusion level"[tiab]

OR "postoperative outcome*"[tiab]

)

EMBASE:

(

'scoliosis'/exp

OR scoliosis:ti,ab

OR 'idiopathic scoliosis':ti,ab

OR 'adolescent idiopathic scoliosis':ti,ab

OR ais:ti,ab

OR 'early onset scoliosis':ti,ab

OR 'neuromuscular scoliosis':ti,ab

OR 'congenital scoliosis':ti,ab

)

AND

(

'spine fusion'/exp

OR 'orthopedic surgery'/exp

OR 'spinal fusion':ti,ab

OR 'posterior spinal fusion':ti,ab

OR psf:ti,ab

OR 'scoliosis correction':ti,ab

OR 'corrective surgery':ti,ab

OR 'scoliosis surgery':ti,ab

OR 'deformity correction':ti,ab

OR 'spine surgery':ti,ab

OR 'posterior fusion':ti,ab

OR instrumentation:ti,ab

)

AND

(

'enhanced recovery':ti,ab

OR eras:ti,ab

OR 'enhanced recovery after surgery':ti,ab

OR 'enhanced recovery pathway':ti,ab

OR 'enhanced recovery protocol':ti,ab

OR 'enhanced recovery program':ti,ab

OR 'rapid recovery':ti,ab

OR 'rapid recovery pathway':ti,ab

OR 'rapid recovery protocol':ti,ab

OR 'accelerated recovery':ti,ab

OR 'accelerated recovery protocol':ti,ab

OR 'accelerated discharge':ti,ab

OR 'accelerated discharge pathway':ti,ab

OR 'clinical pathway'/exp

OR 'clinical pathway':ti,ab

OR 'care pathway':ti,ab

OR 'critical pathway':ti,ab

OR 'perioperative pathway':ti,ab

OR 'perioperative protocol':ti,ab

OR 'perioperative surgical home':ti,ab

OR psh:ti,ab

OR 'multimodal pathway':ti,ab

OR 'multidisciplinary pathway':ti,ab

```
    OR 'standardized perioperative care':ti,ab
    OR 'standardized pathway':ti,ab
    OR 'fast track':ti,ab
    OR 'fast-track':ti,ab
    OR 'fast track surgery':ti,ab
)
AND
(
    'perioperative care'/exp
    OR 'perioperative nursing'/exp
    OR 'nursing care'/exp
    OR 'postoperative care'/exp
    OR 'preoperative care'/exp
    OR perioperative:ti,ab
    OR preoperative:ti,ab
    OR postoperative:ti,ab
    OR intraoperative:ti,ab
    OR nurse*:ti,ab
    OR nursing:ti,ab
    OR 'nursing care':ti,ab
```

OR 'perioperative nursing':ti,ab

OR 'patient education':ti,ab

OR 'pain management':ti,ab

OR 'multimodal analgesia':ti,ab

OR mobilization:ti,ab

OR 'early mobilization':ti,ab

OR ambulation:ti,ab

OR nutrition:ti,ab

OR feeding:ti,ab

OR discharge:ti,ab

OR 'discharge planning':ti,ab

OR 'catheter removal':ti,ab

OR 'drain removal':ti,ab

OR rehabilitation:ti,ab

)

AND

(

'postoperative complication'/exp

OR 'hospital readmission'/exp

OR 'hospital stay'/exp

OR 'operation duration'/exp

OR complication*:ti,ab

OR readmission*:ti,ab

OR 'length of stay':ti,ab

OR los:ti,ab

OR 'hospital stay':ti,ab

OR 'operative time':ti,ab

OR 'operating time':ti,ab

OR 'surgical time':ti,ab

OR 'duration of surgery':ti,ab

OR fusion:ti,ab

OR 'fusion level':ti,ab

OR 'levels fused':ti,ab

OR 'spinal fusion level':ti,ab

OR 'postoperative outcome*':ti,ab

)

CINAHL:

(

(MH "Scoliosis+")

OR TI (scoliosis OR "idiopathic scoliosis" OR "adolescent idiopathic scoliosis" OR AIS OR "early onset scoliosis" OR "neuromuscular scoliosis" OR "congenital scoliosis")

OR AB (scoliosis OR "idiopathic scoliosis" OR "adolescent idiopathic scoliosis" OR AIS OR "early onset scoliosis" OR "neuromuscular scoliosis" OR "congenital scoliosis")

)

AND

(

(MH "Spinal Fusion+")

OR (MH "Orthopedic Surgery+")

OR TI ("spinal fusion" OR "posterior spinal fusion" OR PSF OR "scoliosis correction" OR "corrective surgery" OR "scoliosis surgery" OR "deformity correction" OR "spine surgery" OR "posterior fusion" OR instrumentation)

OR AB ("spinal fusion" OR "posterior spinal fusion" OR PSF OR "scoliosis correction" OR "corrective surgery" OR "scoliosis surgery" OR "deformity correction" OR "spine surgery" OR "posterior fusion" OR instrumentation)

)

AND

(

TI ("enhanced recovery after surgery" OR ERAS OR "enhanced recovery" OR "enhanced recovery pathway" OR "enhanced recovery protocol" OR "enhanced recovery program" OR "rapid recovery" OR "rapid recovery pathway" OR "rapid recovery protocol" OR "accelerated recovery" OR "accelerated recovery protocol" OR "accelerated discharge" OR "accelerated discharge pathway" OR "clinical pathway" OR "care pathway" OR "critical pathway" OR "perioperative pathway" OR "perioperative protocol" OR "perioperative surgical home" OR PSH OR "multimodal pathway" OR "multidisciplinary pathway" OR "standardized perioperative care" OR "standardized pathway" OR "fast track" OR "fast-track" OR "fast track surgery")

OR AB ("enhanced recovery after surgery" OR ERAS OR "enhanced recovery" OR "enhanced recovery pathway" OR "enhanced recovery protocol" OR "enhanced recovery program" OR "rapid recovery" OR "rapid recovery pathway" OR "rapid recovery protocol" OR "accelerated recovery" OR "accelerated recovery protocol" OR "accelerated discharge" OR "accelerated discharge pathway" OR "clinical pathway" OR "care pathway" OR "critical pathway" OR "perioperative pathway" OR "perioperative protocol" OR "perioperative surgical home" OR PSH OR "multimodal pathway" OR "multidisciplinary pathway" OR "standardized perioperative care" OR "standardized pathway" OR "fast track" OR "fast-track" OR "fast track surgery")

)

AND

(

(MH "Perioperative Care+")

OR (MH "Perioperative Nursing")

OR (MH "Nursing Care")

OR (MH "Preoperative Care")

OR (MH "Postoperative Care")

OR TI (perioperative OR preoperative OR postoperative OR intraoperative OR nurse* OR nursing OR "nursing care" OR "perioperative nursing" OR "patient education" OR "pain management" OR "multimodal analgesia" OR mobilization OR "early mobilization" OR ambulation OR nutrition OR feeding OR discharge OR "discharge planning" OR "catheter removal" OR "drain removal" OR rehabilitation)

OR AB (perioperative OR preoperative OR postoperative OR intraoperative OR nurse* OR nursing OR "nursing care" OR "perioperative nursing" OR "patient education" OR "pain management" OR "multimodal analgesia" OR mobilization OR "early mobilization" OR ambulation OR nutrition OR feeding OR discharge OR "discharge planning" OR "catheter removal" OR "drain removal" OR rehabilitation)

)

AND

(

(MH "Postoperative Complications+")

OR (MH "Patient Readmission")

OR (MH "Length of Stay")

OR TI (complication* OR readmission* OR "length of stay" OR LOS OR "hospital stay" OR "operative time" OR "operating time" OR "surgical time" OR "duration of surgery" OR fusion OR "fusion level" OR "levels fused" OR "spinal fusion level" OR

"postoperative outcome*")

OR AB (complication* OR readmission* OR "length of stay" OR LOS OR "hospital stay" OR "operative time" OR "operating time" OR "surgical time" OR "duration of surgery" OR fusion OR "fusion level" OR "levels fused" OR "spinal fusion level" OR "postoperative outcome*")

)

Scopus:

TITLE-ABS-KEY

(

scoliosis

OR "idiopathic scoliosis"

OR "adolescent idiopathic scoliosis"

OR AIS

OR "early onset scoliosis"

OR "neuromuscular scoliosis"

OR "congenital scoliosis"

)

AND

TITLE-ABS-KEY

(

"spinal fusion"

OR "posterior spinal fusion"

OR PSF

OR "scoliosis correction"

OR "corrective surgery"

OR "scoliosis surgery"

OR "deformity correction"

OR "spine surgery"

OR "posterior fusion"

OR instrumentation

)

AND

TITLE-ABS-KEY

(

"enhanced recovery after surgery"

OR ERAS

OR "enhanced recovery"

OR "enhanced recovery pathway"

OR "enhanced recovery protocol"

OR "enhanced recovery program"

OR "rapid recovery"

OR "rapid recovery pathway"

OR "rapid recovery protocol"

OR "accelerated recovery"

OR "accelerated recovery protocol"

OR "accelerated discharge"

OR "accelerated discharge pathway"

OR "accelerated protocol"

OR "accelerated recovery protocol"

OR "clinical pathway"

OR "care pathway"

OR "critical pathway"

OR "perioperative pathway"

OR "perioperative protocol"

OR "perioperative surgical home"

OR PSH

OR "multimodal pathway"

OR "multidisciplinary pathway"

OR "standardized perioperative care"

OR "standardized pathway"

OR "fast track"

OR "fast-track"

OR "fast track surgery"

)

AND

TITLE-ABS-KEY

(

perioperative

OR preoperative

OR postoperative

OR intraoperative

OR nurse*

OR nursing

OR "nursing care"

OR "perioperative nursing"

OR "patient education"

OR "pain management"

OR "multimodal analgesia"

OR mobilization

OR "early mobilization"

OR ambulation

OR nutrition

OR feeding

OR discharge

OR "discharge planning"

OR "catheter removal"

OR "drain removal"

OR rehabilitation

)

AND

TITLE-ABS-KEY

(

complication*

OR readmission*

OR "length of stay"

OR LOS

OR "hospital stay"

OR "operative time"

OR "operating time"

OR "surgical time"

OR "duration of surgery"

OR fusion

OR "fusion level"

OR "levels fused"

OR "spinal fusion level"

OR "postoperative outcome*"

)

Web of Science:

TS=(

scoliosis

OR "idiopathic scoliosis"

OR "adolescent idiopathic scoliosis"

OR AIS

OR "early onset scoliosis"

OR "neuromuscular scoliosis"

OR "congenital scoliosis"

)

AND TS=(

"spinal fusion"

OR "posterior spinal fusion"

OR PSF

OR "scoliosis correction"

OR "corrective surgery"

OR "scoliosis surgery"

OR "deformity correction"

OR "spine surgery"

OR "posterior fusion"

OR instrumentation

)

AND TS=(

"enhanced recovery after surgery"

OR ERAS

OR "enhanced recovery"

OR "enhanced recovery pathway"

OR "enhanced recovery protocol"

OR "enhanced recovery program"

OR "rapid recovery"

OR "rapid recovery pathway"

OR "rapid recovery protocol"

OR "accelerated recovery"

OR "accelerated recovery protocol"

OR "accelerated discharge"

OR "accelerated discharge pathway"

OR "accelerated protocol"

OR "clinical pathway"

OR "care pathway"

OR "critical pathway"

OR "perioperative pathway"

OR "perioperative protocol"

OR "perioperative surgical home"

OR PSH

OR "multimodal pathway"

OR "multidisciplinary pathway"

OR "standardized perioperative care"

OR "standardized pathway"

OR "fast track"

OR "fast-track"

OR "fast track surgery"

)

AND TS=(

perioperative

OR preoperative

OR postoperative

OR intraoperative

OR nurse*

OR nursing

OR "nursing care"

OR "perioperative nursing"

OR "patient education"

OR "pain management"

OR "multimodal analgesia"

OR mobilization

OR "early mobilization"

OR ambulation

OR nutrition

OR feeding

OR discharge

OR "discharge planning"

OR "catheter removal"

OR "drain removal"

OR rehabilitation

)

AND TS=(

complication*

OR readmission*

OR "length of stay"

OR LOS

OR "hospital stay"

OR "operative time"

OR "operating time"

OR "surgical time"

OR "duration of surgery"

OR fusion

OR "fusion level"

OR "levels fused"

OR "spinal fusion level"

OR "postoperative outcome*"

)

Cochrane library:

(

MeSH descriptor: [Scoliosis] explode all trees

OR scoliosis:ti,ab,kw

OR "idiopathic scoliosis":ti,ab,kw

OR "adolescent idiopathic scoliosis":ti,ab,kw

OR AIS:ti,ab,kw

OR "early onset scoliosis":ti,ab,kw

OR "neuromuscular scoliosis":ti,ab,kw

OR "congenital scoliosis":ti,ab,kw

)

AND

(

MeSH descriptor: [Spinal Fusion] explode all trees

OR "spinal fusion":ti,ab,kw

OR "posterior spinal fusion":ti,ab,kw

OR PSF:ti,ab,kw

OR "scoliosis correction":ti,ab,kw

OR "corrective surgery":ti,ab,kw

OR "scoliosis surgery":ti,ab,kw

OR "deformity correction":ti,ab,kw

OR "spine surgery":ti,ab,kw

OR "posterior fusion":ti,ab,kw

OR instrumentation:ti,ab,kw

)

AND

(

"enhanced recovery after surgery":ti,ab,kw

OR ERAS:ti,ab,kw

OR "enhanced recovery":ti,ab,kw

OR "enhanced recovery pathway":ti,ab,kw

OR "enhanced recovery protocol":ti,ab,kw

OR "enhanced recovery program":ti,ab,kw

OR "rapid recovery":ti,ab,kw

OR "rapid recovery pathway":ti,ab,kw

OR "rapid recovery protocol":ti,ab,kw

OR "accelerated recovery":ti,ab,kw

OR "accelerated recovery protocol":ti,ab,kw

OR "accelerated discharge":ti,ab,kw

OR "accelerated discharge pathway":ti,ab,kw

OR "accelerated protocol":ti,ab,kw

OR "clinical pathway":ti,ab,kw

OR "care pathway":ti,ab,kw

OR "critical pathway":ti,ab,kw

OR "perioperative pathway":ti,ab,kw

OR "perioperative protocol":ti,ab,kw

OR "perioperative surgical home":ti,ab,kw

OR PSH:ti,ab,kw

OR "multimodal pathway":ti,ab,kw

OR "multidisciplinary pathway":ti,ab,kw

OR "standardized perioperative care":ti,ab,kw

OR "standardized pathway":ti,ab,kw

OR "fast track":ti,ab,kw

OR "fast-track":ti,ab,kw

OR "fast track surgery":ti,ab,kw

)

AND

(

MeSH descriptor: [Perioperative Care] explode all trees

OR MeSH descriptor: [Nursing Care] explode all trees

OR MeSH descriptor: [Postoperative Care] explode all trees

OR MeSH descriptor: [Preoperative Care] explode all trees

OR perioperative:ti,ab,kw

OR preoperative:ti,ab,kw

OR postoperative:ti,ab,kw

OR intraoperative:ti,ab,kw

OR nurse*:ti,ab,kw

OR nursing:ti,ab,kw

OR "nursing care":ti,ab,kw

OR "perioperative nursing":ti,ab,kw

OR "patient education":ti,ab,kw

OR "pain management":ti,ab,kw

OR "multimodal analgesia":ti,ab,kw

OR mobilization:ti,ab,kw

OR "early mobilization":ti,ab,kw

OR ambulation:ti,ab,kw

OR nutrition:ti,ab,kw

OR feeding:ti,ab,kw

OR discharge:ti,ab,kw

OR "discharge planning":ti,ab,kw

OR "catheter removal":ti,ab,kw

OR "drain removal":ti,ab,kw

OR rehabilitation:ti,ab,kw

)

AND

(

MeSH descriptor: [Postoperative Complications] explode all trees

OR MeSH descriptor: [Patient Readmission] explode all trees

OR MeSH descriptor: [Length of Stay] explode all trees

OR complication*:ti,ab,kw

OR readmission*:ti,ab,kw

OR "length of stay":ti,ab,kw

OR LOS:ti,ab,kw

OR "hospital stay":ti,ab,kw

OR "operative time":ti,ab,kw

OR "operating time":ti,ab,kw

OR "surgical time":ti,ab,kw

OR "duration of surgery":ti,ab,kw

OR fusion:ti,ab,kw

OR "fusion level":ti,ab,kw

OR "levels fused":ti,ab,kw

OR "spinal fusion level":ti,ab,kw

OR "postoperative outcome*":ti,ab,kw

)
